# The psychometric properties of the Bangla Posttraumatic Stress Disorder Checklist for DSM-5 (PCL-5): preliminary reports from a large-scale validation study

**DOI:** 10.1186/s12888-022-03920-4

**Published:** 2022-04-20

**Authors:** Md. Saiful Islam, Most. Zannatul Ferdous, Md. Safaet Hossain Sujan, Rafia Tasnim, Jakir Hossain Bhuiyan Masud, Sourav Kundu, Abu Syed Md. Mosaddek, M. Shahabuddin K. Choudhuri, Ibrahim A. Kira, David Gozal

**Affiliations:** 1 Department of Public Health and Informatics, Jahangirnagar University, Savar, Dhaka-1342 Bangladesh; 2 Centre for Advanced Research Excellence in Public Health, Savar, Dhaka-1342 Bangladesh; 3 Quest Bangladesh Biomedical Research Center, Lalmatia, Dhaka-1207 Bangladesh; 4 Public Health Informatics Foundation (PHIF), Mirpur, Dhaka-1216 Bangladesh; 5 Advanced Institute of Industrial Technology, Shinagawa City, Tokyo, 140-0011 Japan; 6 Department of Pharmacology, Uttara Adhunik Medical College, Uttara, Dhaka-1230 Bangladesh; 7 Department of Pharmacy, Jahangirnagar University, Savar, Dhaka-1342 Bangladesh; 8 United States Pharmacopeial Convention (USP) Herbal Medicines Compendium South Asia Expert Panel Member, New Delhi, India; 9 Center for Cumulative Trauma Studies, Stone Mountain, GA USA; 10 Affiliate of Center for Stress, Trauma and Resiliency, Georgia State University, Atlanta, GA USA; 11 Department of Child Health and the Child Health Research Institute, The University of Missouri School of Medicine, Columbia, MO 65201 USA

**Keywords:** PTSD, PCL-5, Bangla, Depression, Confirmatory factor analysis

## Abstract

**Background:**

The Posttraumatic Stress Disorder Checklist (PCL-5) is the most widely used screening tool in assessing posttraumatic stress disorder symptoms, based on the Diagnostic and Statistical Manual of Mental disorders (DSM-5) criteria. This study aimed to evaluate the psychometric properties of the newly translated Bangla PCL-5.

**Methods:**

A cross-sectional survey was carried out among 10,605 individuals (61.0% male; mean age: 23.6 ± 5.5 [13–71 years]) during May and June 2020, several months after the onset of the COVID-19 outbreak in Bangladesh. The survey included the Bangla PCL-5 and the PHQ-9 depression scale. We used confirmatory factor analysis to test the four-factor DSM-5 model, the six-factor Anhedonia model, and the seven-factor hybrid model.

**Results:**

The Bangla PCL-5 displayed adequate internal consistency (Cronbach’s alpha = 0.90). The Bangla PCL-5 score was significantly correlated with scores of the PHQ-9 depression scale, confirming strong convergent validity. Confirmatory factor analyses indicated the models had a good fit to the data, including the four-factor DSM-5 model, the six-factor Anhedonia model, and the seven-factor hybrid model. Overall, the seven-factor hybrid model exhibited the best fit to the data.

**Conclusions:**

The Bangla PCL-5 appears to be a valid and reliable psychometric screening tool that may be employed in the prospective evaluation of posttraumatic stress disorder in Bangladesh.

**Supplementary Information:**

The online version contains supplementary material available at 10.1186/s12888-022-03920-4.

## Introduction

Bangladesh, a low-middle-income nation with a high population density, is inordinately vulnerable to adverse ecological events due to its geographic location [1]. Indeed, Bangladesh is ranked 9^th^ in the world among the worst affected countries in the 2017 Climate Risk Index, and has consistently ranked in the top 10 on the Long-Term Climate Risk Index for the last two decades [2]. Every year, the country will experience a variety of natural disasters, such as floods, hurricanes, and cyclones, with their attendant consequences of loss of human lives and property. These naturally occurring disasters predispose the general population to an inordinate burden of psychosocial consequences, including acute stress disorder, depression, generalized anxiety disorders, posttraumatic stress disorder (PTSD), sleep disorders, and suicidal ideation [3]. Currently, systematic detection and interventions targeting the psychosocial morbidities of such recurring disasters are not undertaken due to the lack of a robust language-appropriate instrument to assess PTSD particularly in the context of epidemiological surveillance.

PTSD is a common psychiatric condition associated with a stressful experience, and has emerged as a major burden to those affected and to society [4]. Troubles with sleeping, somatic chronic pain, depression, drug abuse, adverse interpersonal relations, and reduced overall wellness are characteristic of PTSD [5, 6]. PTSD follows traumatic events characterized by a typical intrusion symptom pattern, persistence of trauma, avoidance, physiological and emotional numbness, and hypersensitivity [7].

The Posttraumatic Stress Disorder Checklist (PCL) has long been the leading self-reported instrument for assessing PTSD symptoms [8, 9]. Since the popularization of the most recent edition of the Diagnostic and Statistical Manual of Mental Disorders (DSM-5), the PCL has been updated to incorporate additional symptoms and to conform with the four-factor PTSD conceptualization of the DSM (PTSD Checklist for DSM-5 [PCL-5]) and its associated symptom clusters: re-experiencing, avoidance, negative changes in cognition and mood, and increased arousal and reactivity [9, 10]. This transition from the previously outlined three-factor PTSD model in DSM-IV [11], is based on a substantial body of empirical data indicating that this four-factor model better fits the composition of PTSD symptomatology [12, 13].

The PCL-5 [14] consists of 20 items corresponding to the 20 criteria for PTSD outlined in the DSM-5, and includes 4 subscales referring to the 4 symptom clusters mentioned above. It is de facto a modified version of the PCL-4 containing 17 items and three subscales referring to the former three symptom clusters of the DSM-IV [8]. Earlier research on the psychometric properties of the PCL-5 has been promising. In a study of college students, the PCL-5 illustrated positive and significant correlation with depression (convergent validity), proper test–retest reliability, and divergent validity [15]. Such findings are comparable to the psychometric findings in previous versions of the measure [16] and suggest that the PCL-5 has the same psychometric rigor as the previous versions. A PCL-5 cut-off score of 31 to 33 points has been suggested to assess PTSD, and exhibits 88% sensitivity, and 69% specificity [17].

Earlier versions of the PCL were available in multiple languages [18]. However, as far as we know, the PCL-5 is still available in only a few, limited number of languages [9, 19–24]. The PCL-5 exhibited excellent internal consistency for each language including Chinese (α = 0.91), Dutch (α = 0.93), English (α = 0.95), French (α = 0.94), German (α = 0.95), Tagalog (α = 0.95), Turkish (α = 0.94), and Swedish (α = 0.90) [9, 19–24]. For convergent validity, earlier studies reported high correlations ranging from 0.70 to 0.77 between PTSD and depressive symptoms when using the PCL-5 and the Patient Health Questionnaire (PHQ-9) instruments [17, 23, 24]. Likewise, significant correlations (*r* = 0.60–0.65) were also reported using PCL-5 and other screening instruments for depression (e.g., Center for Epidemiological Studies-Depression Scale [CES-D], Beck Depression Inventory [BDI], Depression Hopkins Symptom Checklist [DHSC], and Montgomery-Asberg Depression Rating Scale [MADRS]) [9, 20, 21, 25]. Despite the fact that the DSM-5 proposed a four-factor model of PTSD (i.e., Re-experiencing [B1-B5], Avoidance [C1-C2], Negative alterations in cognitions and mood [D1-D7], and Alterations in arousal and reactivity [E1-E6]), several studies using confirmatory factor analysis (CFA) found that the DSM-5 model is not suitable for the population studied [15, 26, 27]. Current literature suggests that PTSD can be defined by inclusion of numerous factors: for example, the Anhedonia model, proposed by Liu et al., includes six factors of PTSD (i.e., Re-experiencing [B1-B5], Avoidance [C1-C2], Negative affect [D1-D4], Anhedonia [E1-E3], Dysphoric arousal [H1-H2], Anxious arousal [E1-E2], and Dysphoric arousal [H3-H4]) [26], while Armour et al. proposed a seven-factor hybrid model for PTSD (i.e., Re-experiencing [B1-B5], Avoidance [C1-C2], Negative affect [D1-D4], Anhedonia [E1-E3], Externalizing behaviors [F1-F2], Anxious arousal [G1-G2], and Dysphoric arousal [H1-H2]) [27].

COVID-19 was first reported in Bangladesh on March 8^th^, 2020 [28, 29], and similar to many other countries rapidly propagated, with more than 310,800 people infected and 4,248 deaths as of August 31^st^, 2020 [30, 31]. To reduce the spread of SARS-CoV-2, the Government of Bangladesh imposed strict social isolation, home quarantine, and restricted travel measures starting as of March 26^th^, 2020 [32, 33]. Pandemic-related issues such as spatial distancing, isolation and quarantine, as well as social and economic consequences, have naturally led to a multitude of psychosocial responses, including stress, anger, boredom, fear, frustration, grief, depression, and of course PTSD, [34–37], all of which are common mental health problems that many individuals will experience during and after a crisis [38]. Experiencing or witnessing the suffering imposed by COVID-19 can cause PTSD among survivors, their families, frontline workers, and even the general public [39]. It is further anticipated that the COVID-19 pandemic will result in a high prevalence of psychological problems at the population level, including PTSD [40], and previous studies involving outbreaks of SARS in 2003 [41] and influenza A H1N1 in 2009 [42] have corroborated such assumption.

In the context of COVID-19, this pandemic will likely impose major adverse effects on mental health, and yet there are few studies addressing these issues in Bangladesh, as illustrated by the use of a previously not validated tool in a study that was conducted among the survivors of Rana Plaza collapse [5]. To better address this important problem, the PCL-5 was translated to the Bangla language, and in the current study, we present the validation of this instrument as items of the PCL-5 may now be answered more consistently by participants because the items included refer to the same stressful experience. As indicated above, the PCL-5 is one of the most widely used self-report measures of PTSD [15]. In an earlier study, Islam et al. (2020) suggested the need to conduct a nationwide survey to investigate PTSD symptoms and prevalence during the COVID-19 pandemic [36]. The present study was designed to explore the presence of acute posttraumatic stress symptoms among Bangladeshi people in the several months that have followed the onset of the COVID-19 outbreak in the country, and to ascertain whether the Bangla version of the PCL-5 is suitable for the Bangladeshi cultural framework as a screening instrument.

## Methodology

### Study design and participants

This present research study utilized a cross-sectional study design with a sample of 10,605 individuals and conducted between May and June 2020, i.e., nearly 3 months after the onset of the COVID-19 outbreak in Bangladesh. A self-reported anonymous questionnaire with informed consent was developed and administered in online. The targeted participants were the general population of Bangladesh, who could speak and understand Bangla. Individuals approached who were unwilling to participate were not included.

### Study procedure and adaption of the PCL-5 into Bangla

A convenience sampling technique was implemented, but in light of the pandemic situation, the survey questionnaire was conceived and published in online. The questionnaire took roughly 10 to 15 min to complete. The PCL-5 questionnaire was translated using the widest used guideline [i.e. Beaton et al. (2000)] [43]. Firstly, the questionnaire was translated into Bangla (participants’ language) by 3 expert translators, who after reaching consensus on this final Bangla version, the latter was then back-translated into English (i.e., forward–backward translation) by 3 additional translators. All the copies of the questionnaire were then evaluated and approved by the core research team. A conceptual translation was implemented instead of a literal translation to ensure that the original meaning of an item was preserved while adapting it to the Bangladeshi cultural context.

A pilot test was initially conducted to check the reliability of the questionnaire using 150 participants. Then, using Google Survey Tool and with the help of research assistants selected from different areas of Bangladesh to ensure a high response rate, a web-based survey was carried out.

### Measures

To obtain information from participants, a self-reported survey questionnaire was used containing an informed consent form, and questions concerning socio-demographic and psychometric scales (i.e., the PCL-5, and the PHQ-9) [see questionnaire – Supplementary material].

### Socio-demographic measures

During the survey, socio-demographic data were collected by asking questions regarding age, sex, education, occupation, marital status, type of family (nuclear versus joint/extended), monthly family income, residence (rural versus urban), and smoking habits (yes versus no). The monthly family income was categorized into the following three classes based on their monthly total family income in Bangladeshi Taka (BDT): < 15,000 BDT, 15,000–30,000 BDT, and > 30,000 BDT [33, 44].

### PTSD Checklist for DSM-5 (PCL-5)

The Bangla translated version of the PCL-5 is, exactly as the original English version, a self-reported 20-item scale, and evaluates the presence, severity, and 20 symptoms related to PTSD in the DSM-5 [14]. The scale represents the extent to which an individual is suffering from PTSD. Respondents were asked to fill up the scale in the questionnaire following their feelings in the last month (e.g., “*Repeated, disturbing, and unwanted memories of the stressful experience*”), on a five-point Liker scale, which ranges from 0 to 4: 0 “*Not at all*”, 1 “*A little bit*”, 2 “*Moderately*”, 3 “*Quite a bit*”, and 4 “*Extremely*”. The final score was obtained by summating the 20 items, such that the final score ranges from 0–80. It includes four subscales; i) Re-experience (5 symptoms), ii) Avoidance (2 symptoms), iii) Negative alterations in cognition and mood (7 symptoms), and iv) Alterations in arousal and reactivity (6 symptoms). Moreover, researchers proposed several models by the inclusion of numerous factors: for example, the Anhedonia model, proposed by Liu et al., includes six factors of PTSD (i.e., i) Re-experiencing [5 symptoms], ii) Avoidance [2 symptoms], iii) Negative affect [4 symptoms], iv) Anhedonia [3 symptoms], v) Dysphoric arousal [4 symptoms], and vi) Anxious arousal [2 symptoms]) [26], while Armour et al. proposed a seven-factor hybrid model for PTSD (i.e., i) Re-experiencing [5 symptoms], ii) Avoidance [2 symptoms], iii) Negative affect [4 symptoms], iv) Anhedonia [3 symptoms], v) Externalizing behaviors [2 symptoms], vi) Anxious arousal [2 symptoms], and vi) Dysphoric arousal [2 symptoms]) [27]. In the present study, the psychometric properties of the Bangla PCL-5 scale were evaluated and are presented.

### Patient Health Questionnaire (PHQ-9)

The PHQ-9 is the most widely used self-reported screening tool for assessing the severity of depressive disorders [45]. This scale comprises nine items with a four-point Likert scale ranging from 0 (“*Not at all”*) to 3 (“*Nearly every day”*). Each item refers to problems experienced including issues with sleep, exhaustion, changes in appetite, difficulties with concentration, and suicidal thoughts are measured over the past two weeks (e.g., *“Little interest or pleasure in doing things”*). The level of depression varies into five groups as minimal, mild, moderate, moderately severe, and severe based on scoring 0–4, 5–9, 10–14, 15–19, and 20–27 points, respectively. The present study employed the previously validated Bangla PHQ-9 questionnaire to investigate the level of participants’ depressive disorders [46] which has been extensively used [47–50]. In the present study, the PHQ-9 scale was found to have very good reliability (Cronbach’s alpha = 0.89).

### Statistical analysis

The data were analyzed using Microsoft Excel 2019, IBM SPSS Statistics version 25, and IBM SPSS Amos version 23. Descriptive statistics (e.g., frequencies, percentages, means, standard deviations, etc.) were performed using SPSS software. To investigate the validity and reliability of the Bangla PCL-5, its properties were examined and reported both at item-level and scale level.

### Item-level analysis

For the item-level analysis, means, standard deviations, skewness, and kurtosis were computed. Furthermore, item-total correlations, and Cronbach’s alpha-if item deleted were reported.

### Reliability

The internal consistency of the scale and its subscales were examined using reliability coefficients (i.e., Cronbach’s alpha).

### Structural validity

CFA was executed to evaluate the structural validity of the Bangla PCL-5 instrument using SPSS Amos. The three most popular models were investigated for confirmatory factor analysis including the DSM-5 four-factor model, the six-factor Anhedonia model [26], and the seven-factor hybrid model [27].

In all of the CFA models, the chi-square (χ^2^), Root Mean Square Error of Approximation (RMSEA), Standardized Root Mean Square Residual (SRMR), Comparative Fit Index (CFI), Normed Fit Index (NFI), Tucker Lewis Fit Index (TLI), Goodness of Fit Index (GFI), and Adjusted Goodness of Fit (AGFI) were calculated using SPSS Amos. Thresholds and conventional fit indices were applied to investigate the goodness of fit of the model under statistical analysis: RMSEA (0.05;0.08), SRMR (0.05;0.08), CFI (0.90:0.95), GFI (0.90;0.95), AGFI (0.90;0.95), TLI (0.90;0.95), and NFI (0.90;0.95) [51–54].

Furthermore, the Bayesian information criterion (BIC; [55]) and the Akaike information criterion (AIC; [56]) were estimated to evaluate the model fit of these non-nested models. A BIC difference of 6–10 is considered strong support, and a difference of more than 10 is considered very strong support in favor of the model with the lower value [57]. Relatively lower AIC values are usually considered to support a better-fitting model [55].

### Convergent validity

The convergent validity of the Bangla PCL-5 instrument and its subscales was evaluated by reporting its correlations with the related instrument (i.e., PHQ-9). The average variance extracted (AVE) and composite reliability (CR) were also calculated to examine convergent validity. According to Fornell and Larcker, convergent validity is supported when the values of CR and AVE fall into the following acceptable thresholds: CR > 0.6 and AVE > 0.5 [58].

### Ethical considerations

All procedures of this study were carried out in keeping with the principles of Institutional Research Ethics and The Code of Ethics of the World Medical Association involving human subjects (Declaration of Helsinki). Formal ethics approval was granted by the Ethical Review Committee, Uttara Adhunik Medical College, Uttara, Dhaka-1230, Bangladesh. All data were collected anonymously, and all participants consented to the survey willingly. The consent form was clearly outlined and included i) the purpose and process of the research, ii) aims and objectives of the research, iii) data anonymity and privacy, iv) option to participate in the study, and v) right to withdraw data from studies at any time.

## Results

### General characteristics of participants

Initially, 10,850 respondents have submitted the survey form after obtaining informed consent. Of these, 10,664 (98.3%) respondents completed the entire survey. After eliminating surveys that were completed but had data missing, 10,605 surveys were included in the final analysis. Of which 61.0% were male with an average age of 23.6 ± 5.5 years, ranging from 13 to 71 years. A sizeable portion of respondents were students (75.2%), and unmarried (84.0%), and had a bachelor’s degree level of education (67.9%) (Table 1). Moreover, the majority of the responders came from urban areas (63.1%), lived in nuclear families (78.9%), and had monthly family income above 30,000 BDT (45.3%). A sizeable portion of respondents reported as being non-smokers (84.6%).

**Table 1:** General characteristics of participants (*N* = 10,605)

Categorical variables	n	(%)
**Sex**		
Male	6472	(61.0)
Female	4133	(39.0)
**Marital status**		
Unmarried	8903	(84.0)
Married	1657	(15.6)
Divorced	45	(0.4)
**Educational qualification**		
No academic education	84	(0.8)
Primary (1–5 grades)	51	(0.5)
Secondary (6–10 grades)	281	(2.6)
Intermediate (11–12 grades)	1562	(14.7)
Bachelor	7202	(67.9)
Higher education (above bachelor)	1425	(13.4)
**Occupation**		
Student	7976	(75.2)
Private employee	828	(7.8)
Government employee	342	(3.2)
Housewife	303	(2.9)
Businessman	263	(2.5)
Freelancer	164	(1.5)
Farmer	59	(0.6)
Day laborer	39	(0.4)
Unemployed	478	(4.5)
Retired	22	(0.2)
Doctor	48	(0.5)
Others	83	(0.8)
**Family type**		
Nuclear	8369	(78.9)
Join	2236	(21.1)
**Monthly family income**		
< 15,000 BDT	1983	(18.7)
15,000–30,000 BDT	3817	(36.0)
> 30,000 BDT	4805	(45.3)
**Residence**		
Urban area	6696	(63.1)
Rural area	3909	(36.9)
**Smoking habits**		
Yes	1634	(15.4)
No	8971	(84.6)
**Continuous variables**	**Mean**	**(SD)**
Age	23.77	(5.46)

### Item-level analysis

Table 2 presents the mean, standard deviation, item-total correlation, Cronbach’s alpha of the scale if each item is omitted, as well as Skewness and Kurtosis of each translated PCL-5 item. The item-total correlation (Table 2) contained no negative values, indicating that the items were assessing the same construct. All items yielded Skewness and Kurtosis values within the ± 2.0 range, indicating that they were normally distributed [59].

**Table 2:** Item-level psychometric properties of the Bangla PCL-5

PCL-5 item	Mean (SD)	Median	Item-total correlation	Skewness	Kurtosis	Cronbach's α if Item Deleted
1	2.13 (1.21)	2	0.43	0.04	-1.14	0.89
2	1.02 (1.13)	1	0.54	1.04	0.18	0.89
3	1.95 (1.26)	2	0.43	0.21	-1.15	0.89
4	2.14 (1.3)	2	0.49	0.02	-1.25	0.89
5	0.9 (1.11)	1	0.55	1.16	0.46	0.89
6	1.72 (1.22)	1	0.40	0.32	-0.92	0.90
7	1.68 (1.28)	1	0.39	0.35	-0.97	0.90
8	0.99 (1.13)	1	0.50	1.01	0.08	0.89
9	1.22 (1.27)	1	0.59	0.82	-0.45	0.89
10	1.02 (1.2)	1	0.53	1.01	-0.03	0.89
11	1.48 (1.28)	1	0.56	0.53	-0.83	0.89
12	1.45 (1.26)	1	0.61	0.61	-0.77	0.89
13	1.62 (1.39)	1	0.61	0.44	-1.14	0.89
14	1.15 (1.26)	1	0.59	0.89	-0.36	0.89
15	1.53 (1.34)	1	0.63	0.53	-0.97	0.89
16	0.93 (1.19)	0	0.50	1.12	0.15	0.89
17	1.57 (1.21)	1	0.23	0.40	-0.84	0.90
18	1.23 (1.24)	1	0.63	0.77	-0.52	0.89
19	1.59 (1.34)	1	0.63	0.48	-1.02	0.89
20	1.36 (1.36)	1	0.55	0.65	-0.88	0.89

### Reliability

Cronbach’s alpha for the total score and each of the subscales of the Bangla PCL-5 instrument are presented in Table 3. The coefficients of Cronbach’s alpha were calculated to investigate internal consistency. Cronbach’s alpha for the total PCL-5 was 0.90, indicating excellent internal consistency, which is well beyond the accepted threshold of 0.70 [58, 60].

**Table 3:** The descriptive statistics, and Cronbach’s alpha of each scale/subscale, and correlations among all scales along with subscales

Scales/subscales	Mean (SD)	Item Range	α	1	2	3	4	5	6	7	8	9	10
1. R	8.14 (4.26)	5 (0–20)	0.75	―									
2. A	3.40 (2.10)	2 (0–8)	0.58	0.43*	―								
3. NACM	8.92 (6.18)	7 (0–28)	0.83	0.52*	0.38*	―							
4. AR	8.20 (5.20)	6 (0–24)	0.76	0.54*	0.36*	0.73*	―						
5. NA	4.71 (3.67)	4 (0–16)	0.74	0.47*	0.35*	0.91*	0.64*	―					
6. An	4.21 (3.21)	3 (0–12)	0.76	0.47*	0.32*	0.88*	0.67*	0.61*	―				
7. DA	5.41 (3.95)	4 (0–16)	0.75	0.50*	0.32*	0.73*	0.95*	0.63*	0.69*	―			
8. AA	2.80 (1.92)	2 (0–8)	0.37	0.43*	0.31*	0.45*	0.76*	0.42*	0.39*	0.51*	―		
9. EB	2.46 (2.12)	2 (0–8)	0.58	0.41*	0.27*	0.68*	0.84*	0.59*	0.63*	0.88*	0.47*	―	
10. Total PCL-5	28.66 (14.52)	20 (0–80)	0.90	0.77*	0.56*	0.89*	0.88*	0.80*	0.80*	0.84*	0.64*	0.75*	―
11. Total PHQ-9	9.02 (6.81)	9 (0–27)	0.89	0.44*	0.26*	0.67*	0.67*	0.58*	0.64*	0.70*	0.36*	0.59*	0.69*

*SD* Standard deviation, *α* Cronbach alpha, *R* Re-experiencing, *A* Avoidance, *NACM* Negative alterations in cognitions and mood, *AR* Alterations in arousal and reactivity, *NA* Negative affect, *An* Anhedonia, *DA* Dysphoric arousal, *AA* Anxious arousal, *EB* Externalizing behaviors, *PCL-5* Posttraumatic Stress Disorder Checklist, *PHQ-9* Patient Health Questionnaire; *p < 0.001

### Construct validity

CFA was performed to appraise the structural validity of the Bangla PCL-5 instrument using the DSM-5 four-factor model, the six-factor Anhedonia model, and the seven-factor hybrid model. Each of the CFA models, the Absolute Fit (i.e., χ^2^, RMSEA, SRMR, GFI), and the Incremental Fit (i.e., AGFI, CFI, TLI, NFI) were observed for the model fit estimation (see Table 4). All fitness indexes were satisfactory within their conventional thresholds, which the models displaying an excellent fit to the data. Of these, the seven-factor hybrid model exhibited the best fit to the data (see Table 4). Furthermore, AIC and BIC values also support the seven-factor hybrid model, as this model also exhibited the lowest AIC and BIC values.

**Table 4:** Scale-level psychometric properties of the Bangla PCL-5

Name of index	Index Abbreviation	Four-factor DSM-5 model	Six-factor Anhedonia model	Seven-factor Hybrid model	Level of acceptance
**Absolute Fit**					
Discrepancy chi square	χ^2^ (df)	7905.8* (164)	6243.7* (155)	5967.0* (149)	*p* > 0.05
Root Mean Square Error of Approximation 90% Confidence interval	RMSEA 90% CI	0.08 (0.065–0.068)	0.06 (0.060–0.062)	0.06 (0.059–0.062)	< 0.08
Standardized Root Mean Square Residual	SRMR	0.05	0.05	0.05	< 0.08
The goodness of Fit Index	GFI	0.92	0.93	0.94	> 0.9
**Incremental Fit**					
Adjusted Goodness of Fit	AGFI	0.9	0.91	0.91	> 0.9
Comparative Fit Index	CFI	0.9	0.91	0.92	> 0.9
Tucker-Lewis Index	TLI	0.9	0.9	0.9	> 0.9
Normed Fit Index	NFI	0.9	0.91	0.92	> 0.9
**Information Criteria**					
Akaike Information Criterion	AIC	7997.84	6353.69	6089.01	Lower indicating better fit
Bayesian Information Criterion	BIC	8332.22	6753.49	6532.42	Lower indicating better fit
**Reliability**					
Composite Reliability	CR	0.93	0.93	0.94	> 0.6
Average Variance Extracted	AVE	0.39	0.39	0.43	> 0.5

^*^p < 0.001

**Fig. 1:**
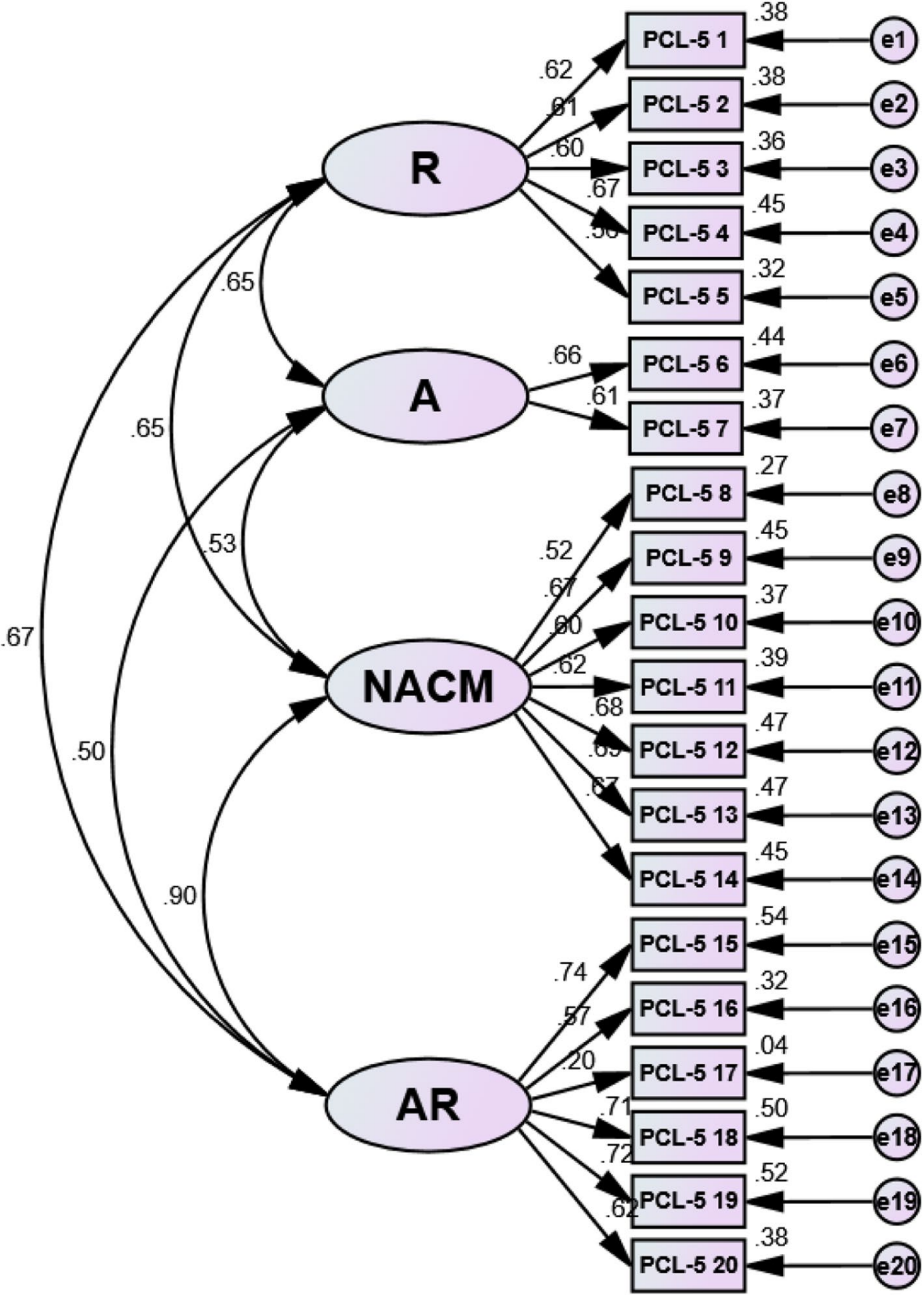
Structural equation modeling (SEM) of the DSM-5 four-factor model. Note: R = Re-experiencing; A = Avoidance; NACM = Negative alterations in cognitions and mood; AR = Alterations in arousal and reactivity

**Fig. 2:**
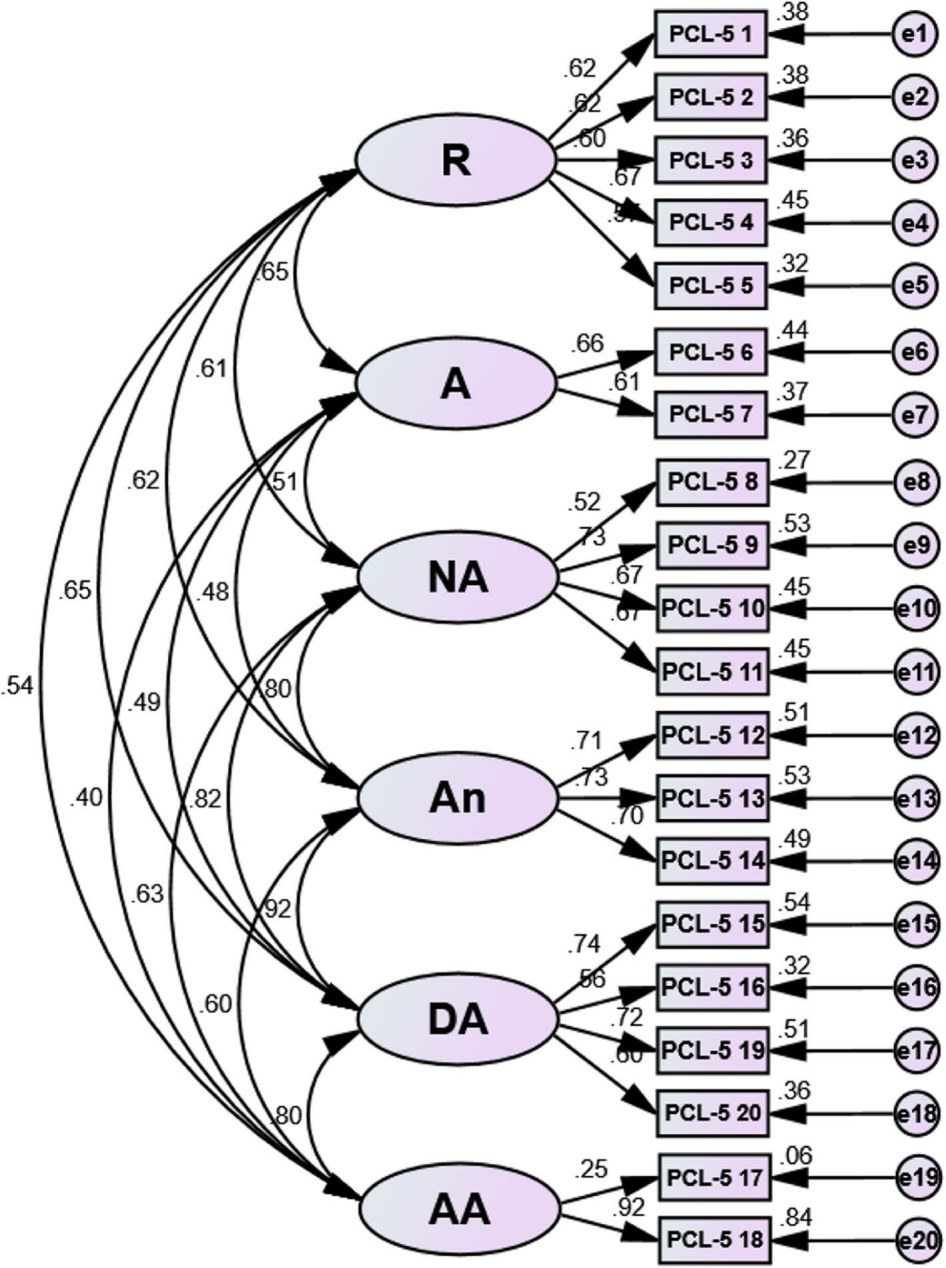
Structural equation modeling (SEM) of the six-factor Anhedonia model. Note: R = Re-experiencing; A = Avoidance; NA = Negative affect; An = Anhedonia; DA = Dysphoric arousal; AA = Anxious arousal

**Fig. 3:**
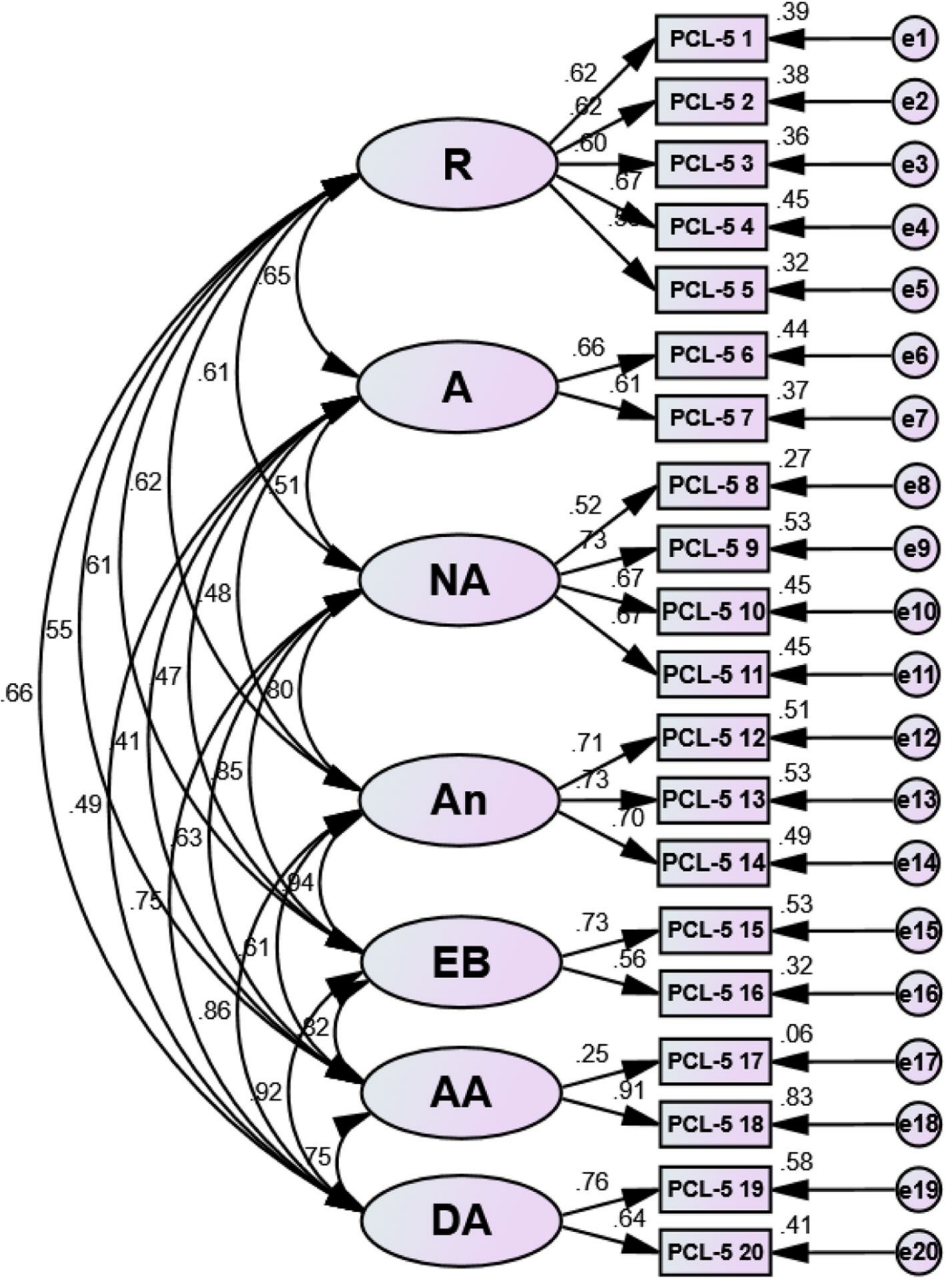
Structural equation modeling (SEM) of the seven-factor hybrid model. Note: R = Re-experiencing; A = Avoidance; NA = Negative affect; An = Anhedonia; EB = Externalizing behaviors; AA = Anxious arousal; DA = Dysphoric arousal

Factor loadings for each model of the Bangla PCL-5 ranged between 0.52 and 0.92 (see Table 5) except item no 17. The acceptability factor was greater than the load value of 0.32 [61]. Structural equation modeling (SEM) revealed a positive correlation between each latent variable in all examined three models – the DSM-5 four-factor model, the six-factor Anhedonia model, and the seven-factor hybrid model, respectively (see ― Figs. 1, 2 and 3), indicating that the items were assessing the same construct.

**Table 5:** Standardized factor loading estimates for confirmatory factor analysis models

DSM-5 symptoms	Four-factor DSM-5 model		Six-factor Anhedonia model		Seven-factor Hybrid model	
	**Factor**	Factor Loads	**Factor**	Factor Loads	**Factor**	Factor Loads
1. Repeated memories	**R**	0.62	**R**	0.62	**R**	0.62
2. Repeated dreams	**R**	0.62	**R**	0.62	**R**	0.62
3. Flashbacks	**R**	0.60	**R**	0.60	**R**	0.60
4. Upset when reminded	**R**	0.68	**R**	0.67	**R**	0.67
5. Physical reaction when reminded	**R**	0.57	**R**	0.57	**R**	0.56
6. Avoidance of thoughts	**A**	0.66	**A**	0.66	**A**	0.66
7. Avoidance of reminders	**A**	0.61	**A**	0.61	**A**	0.61
8. Trouble remembering	**NACM**	0.52	**NA**	0.52	**NA**	0.52
9. Negative beliefs	**NACM**	0.67	**NA**	0.73	**NA**	0.73
10. Blame of self or others	**NACM**	0.60	**NA**	0.67	**NA**	0.67
11. Negative feelings	**NACM**	0.62	**NA**	0.67	**NA**	0.67
12. Loss of interest	**NACM**	0.68	**An**	0.71	**An**	0.71
13. Feeling distant	**NACM**	0.69	**An**	0.73	**An**	0.73
14. Trouble positive feelings	**NACM**	0.67	**An**	0.70	**An**	0.70
15. Irritable behavior	**AR**	0.74	**DA**	0.74	**EB**	0.73
16. Reckless behavior	**AR**	0.57	**DA**	0.56	**EB**	0.56
17. Being super alert	**AR**	0.21	**AA**	0.25	**AA**	0.25
18. Feeling jumpy	**AR**	0.71	**AA**	0.92	**AA**	0.91
19. Difficulty concentrating	**AR**	0.72	**DA**	0.72	**DA**	0.76
20. Trouble sleeping	**AR**	0.62	**DA**	0.60	**DA**	0.64

*R* Re-experiencing, *A* Avoidance, *NACM* Negative alterations in cognitions and mood, *AR* Alterations in arousal and reactivity, *NA* Negative affect, *An* Anhedonia, *DA* Dysphoric arousal, *AA* Anxious arousal, *EB* Externalizing behaviors

### Convergent validity

To evaluate the convergent validity of the Bangla PCL-5 instrument, its total, as well as its subscales correlations with the PHQ-9 instrument, are outlined in Table 3. The correlation between the Bangla PCL-5 and the PHQ-9 generated a significant and positive correlation (*r* = 0.69, *p* < 0.001), confirming strong convergent validity. Furthermore, the correlation between the PCL-5 subscales and PHQ-9 scale yielded a positive correlation in each case (i.e., Re-experiencing: *r* = 0.44; Avoidance: *r* = 0.26; Negative alterations in cognitions and mood: *r* = 0.67; Arousal: *r* = 0.67; Negative affect = 0.58; Anhedonia = 0.64; Dysphoric arousal = 0.70; Anxious arousal = 0.36; Externalizing behaviors = 0.59; *p* < 0.001 for all comparisons).

The values of AVE and CR of the three models ranged between 0.93–0.94, and 0.39–0.43, respectively (see Table 4). The convergent validity is supported if the CR is higher than 0.6, and the AVE is higher than 0.5 for each construct [58]. According to Fornell and Larcker, if the AVE is less than 0.5, but the CR is higher than 0.6, the convergent validity of the construct is still adequate [58].

## Discussion

The present study aimed to translate and validate the Bangla PCL-5 in a large cohort in Bangladesh after at least nearly months from the beginning of the COVID-19 outbreak, and as such enable the use of the most widely used psychometric tool to assess PTSD. Several studies conducted with different cohorts including general population, university students, medical students, slum-dwellers, health workers, and COVID-19 survivors highlighted various mental health problems (e.g., anxiety, depression, panic, stress, suicidal ideation, and behavioral problems such as problematic use of smartphone, internet, social media) in Bangladesh during the pandemic [32, 34, 37, 49, 62–68]. These mental health problems can increase the prevalence of PTSD as a result of experiences related to the COVID-19 pandemic [69]. Several studies corroborated that PTSD was prevalent during the COVID-19 pandemic in other countries, including in China (2.7%-12.8%) [70–72], Saudi Arabia (19.6%-24.8%) [69], Italy (29.5%) [73], Spain (15.8%) [74], and in the USA (31.8%) [75]. Thus, a nationwide study would be desirable to investigate PTSD in Bangladesh. Considering the lack of currently available validated instruments for assessing PTSD, the study findings should contribute to future studies aimed at investigating PTSD in Bangladesh.

The Bangla PCL-5 emerged as psychometrically sound and as a robust instrument since it demonstrated (i) excellent internal consistency and reliability, (ii) construct validity, and (iii) strong convergent validity. The findings suggest that Bangla PCL-5 is a valid and potentially useful tool to assess posttraumatic stress disorder among Bangladeshi people.

The findings revealed excellent internal consistency of the Bangla version of PCL-5, which was similar to previous studies in different languages [9, 20–24]. The Cronbach’s alpha of the subscales of the Bangla PCL-5 was also very similar to the aforementioned studies examining translations into different languages. The inter-item correlation matrix yielded positive values across all items, indicating that these items were assessing the same construct. All items had Skewness and Kurtosis values within the ± 2.0 range, indicating that they were normally distributed [59]. Accordingly, the Bangla PCL-5 showed significant validity at this stage of assessment and performed similarly to previous efforts to translate the instrument in other countries.

The construct validity of the Bangla PCL-5 was also corroborated by confirmatory factor analysis using the DSM-5 four-factor model (i.e., Re-experiencing [B1-B5], Avoidance [C1-C2], Negative alterations in cognitions and mood [D1-D7], and Alterations in arousal and reactivity [E1-E6]) [14], the six-factor Anhedonia model (i.e., Re-experiencing [B1-B5], Avoidance [C1-C2], Negative affect [D1-D4], Anhedonia [E1-E3], Dysphoric arousal [H1-H2], Anxious arousal [E1-E2], and Dysphoric arousal [H3-H4]) [26], and the seven-factor hybrid model (i.e., Re-experiencing [B1-B5], Avoidance [C1-C2], Negative affect [D1-D4], Anhedonia [E1-E3], Externalizing behaviors [F1-F2], Anxious arousal [G1-G2], and Dysphoric arousal [H1-H2]) [27]. Within their conventional thresholds, all fitness indices were highly satisfactory, indicating that the models were an excellent fit to the data. Of these, the seven-factor hybrid model exhibited the best fit (see Table 4).. The findings resonate with the previous studies that reported that the seven-factor hybrid model is the best fitting model in diverse populations, including community population [76, 77], treatment-seeking population [78], university students [9], and military personnel [15, 27, 79]. A more recent study conducted among Chinese healthcare workers during the COVID-19 pandemic also found the seven-factor hybrid model as the best fitting model [19].

The correlation between the PCL-5 and depression (using PHQ-9) generated a significant and positive correlation (*r* = 0.69, *p* < 0.001), confirming strong convergent validity, a finding that has also been similarly reported in previous studies using a similar instrument (Philippines: PCL-5 vs. PHQ-9, *r* = 0.71; [23]; Netherlands: PCL-5 vs. PHQ-9, *r* = 0.72; [24]). A strong correlation obtained between the PCL-5 and depression using different instruments in different languages including English (PCL-5 vs. CES-D, *r* = 0.64) [9], French (PCL-5 vs. CES-D, *r* = 0.62) [9], Turkish (PCL-5 vs. BDI, *r* = 0.64) [20], Arab/Kurdish (PCL-5 vs. DHSC, *r* = 0.65) [25], and Swedish (PCL-5 vs. MADRS, *r* = 0.60) [21], further confirms the robustness of the tool across various translations including Bangla. Moreover, the CR yielded factors ranging (0.93–0.94) for each model, which is well beyond the accepted threshold of 0.60 [58] and supported convergent validity. The AVE obtained ranges of 0.39–0.43 for each model. Of note, and according to Fornell and Larcker, if AVE is less than 0.5, but CR is higher than 0.6, the convergent validity of the construct is still adequate [58].

At last, the utility of PCL-5 in self-report questionnaires that can be administered widely is obviously preferable over other clinical measures such as Clinician-Administered PTSD Scale for DSM-5 (CAPS-5; [80]) that needs to be administered in person and therefore requires different settings and higher resources. CAPS-5, is however, the gold standard for PTSD assessment and is obviously more applicable in clinical studies [80]. Thus, head to head comparisons between PCL-5 and CAPS-5 may be examined in future studies.

### Limitations

Although the psychometric properties of the Bangla PCL-5 were overall satisfactory, there are some potential limitations worthy of commentary. First, compared with face-to-face interviews, self-reporting has limitations including potential multiple biases (e.g., memory recall, social desirability biases). Secondly, the study executed a cross-sectional study design. Longitudinal observation is important, particularly given the potential for posttraumatic experiences and temporal evolution characteristics of this process. Thirdly, this was an online-based survey, so this study was not representative, for example, of those who have limited internet access or may be unwilling to respond via this methodology. Randomized prospective studies could provide potential insights into causation, although these may be complicated to conduct during a pandemic such as COVID-19. Moreover, the study investigated somewhat constrained measures, and did not evaluate other aspects of reliability and validity, such as test–retest reliability, convergent validity (using another PTSD instrument), divergent validity and criterion validity, due to the limited number of test scales available. Likewise, the study did not use other scales to measure PTSD and other psychological symptoms in order to determine the PCL-5's divergent validity. Further investigations are warranted with the inclusion of incremental measures along with their application to clinical settings. Moreover, the exposure to and the number of experienced traumatic events have not been assessed. The study didn’t investigate the sensitivity and specificity of the PCL-5. Further studies should be designed focusing on its sensitivity and specificity (e.g., including traumatized PTSD patients, traumatized non-PTSD patients, clinical controls, and healthy controls).

## Conclusions

To our knowledge, this is the first translation and validation of the PCL-5 into Bangla. We evaluated the comprehensive psychometric properties of this instrument in a large sample while ascertaining the contextual Bangladeshi cultural background setting. Our findings indicate that the Bangla PCL-5 appears to be a robust instrument to screen for the presence of posttraumatic stress disorders among Bangladeshi individuals. In light of the protracted course of the COVID-19 pandemic and the restrictive measures being implemented as well as the economic devastation imposed by this virus, the Bangla PCL-5 instrument will aid in the assessments of posttraumatic stress disorders in Bangladesh and potentially serve as the primary screening tool for further evaluation and treatment of affected individuals. Moreover, due to the lack of divergent validity measures or evaluations of specificity and sensitivity, this study will contribute to the future research on PTSD in Bangladesh.

## Supplementary Information

**Additional file 1**.

## Data Availability

The datasets/ questionnaire generated and/or analyzed during the current study are available from the corresponding author on reasonable request.

## Abbreviations

PCL-5: Posttraumatic Stress Disorder Checklist
DSM-5: Diagnostic and Statistical Manual of Mental disorders
PTSD: Posttraumatic stress disorder
COVID-19: Coronavirus disease-2019
SARS-CoV-2: Severe acute respiratory syndrome coronavirus 2
BDT: Bangladeshi Taka
PHQ-9: Patient Health Questionnaire
ANOVA: Analysis of variance
SPSS: Statistical Package for the Social Sciences
CR: Composite reliability
CFA: Confirmatory factor analysis
SEM: Structural equation modeling
AVE: Average variance extracted
AIC: Akaike Information Criterion
BIC: Bayesian Information Criterion
CAPS-5: Clinician-Administered PTSD Scale for DSM-5
