# Supplementary material for: The psychometric properties of the Bangla Posttraumatic Stress Disorder Checklist for DSM-5 (PCL-5): preliminary reports from a large-scale validation study
Source: BMC Psychiatry. 2022 Apr 20;22:280. doi: 10.1186/s12888-022-03920-4 (PMC9020159; doi:10.1186/s12888-022-03920-4)
Supplement: Supplementary file 1 — Additional file 1. [file 12888_2022_3920_MOESM1_ESM.docx]

**Title page**

**The psychometric properties of the Bangla Posttraumatic Stress Disorder Checklist for DSM-5 (PCL-5): Preliminary reports from a large-scale validation study**

**Author(s):** Md. Saiful Islam1,2,*, Most. Zannatul Ferdous1,3, Md. Safaet Hossain Sujan1,2, Rafia Tasnim1,2, Jakir Hossain Bhuiyan Masud3,4, Sourav Kundu3,5, Abu Syed Md. Mosaddek3,6, M. Shahabuddin K. Choudhuri3,7,8, Ibrahim A. Kira9,10, David Gozal11

**Affiliation(s):**

1 Department of Public Health and Informatics, Jahangirnagar University, Savar, Dhaka-1342, Bangladesh

2 Centre for Advanced Research Excellence in Public Health, Savar, Dhaka-1342, Bangladesh

3 Quest Bangladesh, Lalmatia, Dhaka-1207, Bangladesh

4 Public Health Informatics Foundation (PHIF), Mirpur, Dhaka-1216, Bangladesh

5 Advanced Institute of Industrial Technology, Shinagawa City, Tokyo 140-0011, Japan

6 Department of Pharmacology, Uttara Adhunik Medical College, Uttara, Dhaka-1230, Dhaka, Bangladesh

7 Department of Pharmacy, Jahangirnagar University, Savar, Dhaka-1342, Bangladesh

8 United States Pharmacopeial Convention (USP) Herbal Medicines Compendium South Asia Expert Panel Member, India

9 Center for Cumulative Trauma Studies, Stone Mountain, GA, USA

10 Affiliate of Center for Stress, Trauma and Resiliency, Georgia State University, Atlanta, GA, USA

11 Department of Child Health and the Child Health Research Institute, The University of Missouri School of Medicine, Columbia, MO 65201, USA

**Email address for each author:**

Md. Saiful Islam ([islam.msaiful@outlook.com](mailto:islam.msaiful@outlook.com)), Most. Zannatul Ferdous ([m.zannatul.ferdous@juniv.edu](mailto:m.zannatul.ferdous@juniv.edu)), Md. Safaet Hossain Sujan ([sujanmahmuddphi@gmail.com](mailto:sujanmahmuddphi@gmail.com)), Rafia Tasnim ([tasnimrifa97@gmail.com](mailto:tasnimrifa97@gmail.com)), Jakir Hossain Bhuiyan Masud ([jakirmsd@gmail.com](mailto:jakirmsd@gmail.com)), Sourav Kundu ([sourav@kundu.org](mailto:sourav@kundu.org)), Abu Syed Md. Mosaddek ([drmosaddek1968@gmail.com](mailto:drmosaddek1968@gmail.com)), M. Shahabuddin K. Choudhuri ([mskchoudhuri@juniv.edu](mailto:mskchoudhuri@juniv.edu)), Ibrahim A. Kira ([kiraaref@aol.com](mailto:kiraaref@aol.com)), David Gozal ([gozald@health.missouri.edu](mailto:gozald@health.missouri.edu))

**Corresponding Author:**

**Md. Saiful Islam**

Department of Public Health and Informatics, Jahangirnagar University, Savar, Dhaka-1342, Bangladesh

E-mail: [islam.msaiful@outlook.com](mailto:islam.msaiful@outlook.com) or [saiful@phiju.edu.bd](mailto:saiful@phiju.edu.bd)

Mobile: +8801779439529

ORCID iD: <https://orcid.org/0000-0003-3979-2423>

**The psychometric properties of the Bangla Posttraumatic Stress Disorder Checklist for DSM-5 (PCL-5): Preliminary reports from a large-scale validation study**

Greetings, dear we have started a survey entitled “The psychometric properties of the Bangla Posttraumatic Stress Disorder Checklist for DSM-5 (PCL-5): Preliminary reports from a large-scale validation study”. The results of the study will represent the psychometric properties of the Bangla PCL-5. The survey may take 10-15 minutes. Nowhere in the survey, you won’t be asked for your personal information. All of your information will be kept secret. You have the right to participate or deny, and during the time of participation, you can withdraw yourself from responding. The study will not be benefitted you by money or other compensations but the outcome of the study may consider by the policymakers and take initiative for people with posttraumatic stress disorder (PSTD) in Bangladesh. As there is no validated instrument in the Bangla language to measure PSTD, the findings will be helpful to the researchers in respective filed to conduct epidemiological investigations. The participants below 18 years need to be provided informed consent from a parent and/or legal guardian.

Are you willing to participate in the survey?

1. Yes 2. No

| **Section A: Socio-demographic measures** | |
| --- | --- |
| Age | …………………………….. |
| Sex | 1. Male  2. Female |
| Educational qualification | 1. No academic education  2. Primary (1-5 grades)  3. Secondary (6-10 grades)  4. Intermediate (11-12 grades)  5. Bachelor  6. Higher education (above bachelor) |
| Occupation | 1. Student  2. Private employee  3. Government employee  4. Housewife  5. Businessman  6. Freelancer  7. Farmer  8. Day laborer  9. Unemployed  10. Retired  11. Doctor  12. Others…………….. |
| Marital status | 1. Unmarried  2. Married  3. Divorced |
| Family type | 1. Nuclear  2. Join |
| Monthly family income | 1. <15,000 Bangladeshi Taka (BDT)  2. 15,000-30,000  3. >30,000 |
| Residence | 1. Urban area  2. Rural area |
| Smoking habits | 1. Yes  2. No |

| **Section B: Patient Health Questionnaire (PHQ-9)** | | | | | | | | | |
| --- | --- | --- | --- | --- | --- | --- | --- | --- | --- |
| **Over the last 2 weeks, how often have you been bothered by any of the following problems?**  **(Use “✔” to indicate your answer)** | | **Not at all** | | **Several days** | | **More than half the days** | | **Nearly every day** | |
| 1. Little interest or pleasure in doing things | | 0 | | 1 | | 2 | | 3 | |
| 2. Feeling down, depressed, or hopeless | | 0 | | 1 | | 2 | | 3 | |
| 3. Trouble falling or staying asleep, or sleeping too much | | 0 | | 1 | | 2 | | 3 | |
| 4. Feeling tired or having little energy | | 0 | | 1 | | 2 | | 3 | |
| 5. Poor appetite or overeating | | 0 | | 1 | | 2 | | 3 | |
| 6. Feeling bad about yourself — or that you are a failure or have let yourself or your family down | | 0 | | 1 | | 2 | | 3 | |
| 7. Trouble concentrating on things, such as reading the newspaper or watching television | | 0 | | 1 | | 2 | | 3 | |
| 8. Moving or speaking so slowly that other people could have noticed? Or the opposite — being so fidgety or restless that you have been moving around a lot more than usual | | 0 | | 1 | | 2 | | 3 | |
| 9. Thoughts that you would be better off dead or of hurting yourself in some way | | 0 | | 1 | | 2 | | 3 | |
| **Section C: PTSD Checklist for DSM-5 (PCL-5)** | | | | | | | | | |
| **Instructions: Below is a list of problems that people sometimes have in response to a very stressful experience. Please read each problem carefully and then circle one of the numbers to the right to indicate how much you have been bothered by that problem in the past month.** | | | | | | | | | |
| **In the past month, how much were you bothered by:** | **Not at all** | | **A little bit** | | **Moderately** | | **Quite a bit** | | **Extremely** |
| 1. Repeated, disturbing, and unwanted memories of the stressful experience? | 0 | | 1 | | 2 | | 3 | | 4 |
| 2. Repeated, disturbing dreams of the stressful experience? | 0 | | 1 | | 2 | | 3 | | 4 |
| 3. Suddenly feeling or acting as if the stressful experience were actually happening again (as if you were actually back there reliving it)? | 0 | | 1 | | 2 | | 3 | | 4 |
| 4. Feeling very upset when something reminded you of the stressful experience? | 0 | | 1 | | 2 | | 3 | | 4 |
| 5. Having strong physical reactions when something reminded you of the stressful experience (for example, heart pounding, trouble breathing, sweating)? | 0 | | 1 | | 2 | | 3 | | 4 |
| 6. Avoiding memories, thoughts, or feelings related to the stressful experience? | 0 | | 1 | | 2 | | 3 | | 4 |
| 7. Avoiding external reminders of the stressful experience (for example, people, places, conversations, activities, objects, or situations)? | 0 | | 1 | | 2 | | 3 | | 4 |
| 8. Trouble remembering important parts of the stressful experience? | 0 | | 1 | | 2 | | 3 | | 4 |
| 9. Having strong negative beliefs about yourself, other people, or the world (for example, having thoughts such as: I am bad, there is something seriously wrong with me, no one can be trusted, the world is completely dangerous)? | 0 | | 1 | | 2 | | 3 | | 4 |
| 10. Blaming yourself or someone else for the stressful experience or what happened after it? | 0 | | 1 | | 2 | | 3 | | 4 |
| 11. Having strong negative feelings such as fear, horror, anger, guilt, or shame? | 0 | | 1 | | 2 | | 3 | | 4 |
| 12. Loss of interest in activities that you used to enjoy? | 0 | | 1 | | 2 | | 3 | | 4 |
| 13. Feeling distant or cut off from other people? | 0 | | 1 | | 2 | | 3 | | 4 |
| 14. Trouble experiencing positive feelings (for example, being unable to feel happiness or have loving feelings for people close to you)? | 0 | | 1 | | 2 | | 3 | | 4 |
| 15. Irritable behavior, angry outbursts, or acting aggressively? | 0 | | 1 | | 2 | | 3 | | 4 |
| 16. Taking too many risks or doing things that could cause you harm? | 0 | | 1 | | 2 | | 3 | | 4 |
| 17. Being “superalert” or watchful or on guard? | 0 | | 1 | | 2 | | 3 | | 4 |
| 18. Feeling jumpy or easily startled? | 0 | | 1 | | 2 | | 3 | | 4 |
| 19. Having difficulty concentrating? | 0 | | 1 | | 2 | | 3 | | 4 |
| 20. Trouble falling or staying asleep? | 0 | | 1 | | 2 | | 3 | | 4 |
